# Citrate, low pH and amino acid limitation induce citrate utilization in *Lactococcus lactis* biovar diacetylactis

**DOI:** 10.1111/1751-7915.13031

**Published:** 2017-12-07

**Authors:** Oscar van Mastrigt, Emma E. Mager, Casper Jamin, Tjakko Abee, Eddy J. Smid

**Affiliations:** ^1^ Laboratory of Food Microbiology Wageningen University and Research Wageningen The Netherlands

## Abstract

In *Lactococcus lactis* subsp. *lactis* biovar diacetylactis, citrate transport is facilitated by the plasmid‐encoded citrate permease (CitP). In this work, we analysed regulation of citrate utilization by pH, nutrient limitation and the presence of citrate at four different levels: (i) plasmid copy number, (ii) *citP* transcription, (iii) *citP* mRNA processing and (iv) citrate utilization capacity. Citrate was supplied as cosubstrate together with lactose. The *citP* gene is known to be induced in cells grown at low pH. However, we demonstrated that transcription of *citP* was even higher in the presence of citrate (3.8‐fold compared with 2.0‐fold). The effect of citrate has been overlooked by other researchers because they determined the effect of citrate using M17 medium, which already contains 0.80 ± 0.07 mM citrate. The plasmid copy number increased in cells grown under amino acid limitation (1.6‐fold) and/or at low pH (1.4‐fold). No significant differences in *citP* mRNA processing were found. Citrate utilization rates increased from approximately 1 to 65 μmol min^−1^ gDW^−1^ from lowest to highest *citP* expression. Acetoin formation increased during growth in an acidic environment due to induction of the acetoin pathway. Quantification of the relative contributions allowed us to construct a model for regulation of citrate utilization in *L. lactis* biovar diacetylactis. This knowledge will help to select conditions to improve flavour formation from citrate.

## Introduction

Citrate is an important precursor for flavour formation in dairy fermentations. In contrast to lactose which can be consumed by many lactic acid bacteria, citrate can be degraded only by particular lactic acid bacteria, such as *Lactococcus lactis* biovar diacetylactis. These bacteria convert citrate via oxaloacetate into pyruvate (Harvey and Collins, 1961), which can be further converted into acetoin and diacetyl. The latter two compounds are responsible for the buttery flavour of dairy products. The intermediate pyruvate is a central metabolite in the metabolism of lactic acid bacteria and is also the end‐product of glycolysis. However, production of acetoin and diacetyl is mainly linked to citrate metabolism because no reducing equivalents are produced during citrate degradation (Collins, 1972; Hugenholtz, 1993).

*Lactococcus lactis* biovar diacetylactis is one of the main species in dairy starter cultures that degrades citrate. In this bacterium, the rate of citrate utilization is limited by the rate of citrate transport across the cell membrane (Harvey and Collins, 1962), which is facilitated by a citrate permease. This permease transports divalent citrate either in symport with a proton or in exchange for monovalent L‐lactate (Cachon *et al*., 1995; Marty‐Teysset *et al*., 1995, 1996; Bandell *et al*., 1998). Divalent citrate is most abundant around pH 5.5, which agrees with the observed optimum pH for citrate utilization (Smith *et al*., 1992).

The gene encoding the citrate permease (*citP*) is located on a plasmid in *L. lactis* biovar diacetylactis (Kempler and McKay, 1979a,b, 1981). In contrast, all other lactococcal genes required for citrate degradation are located on the chromosome (Martín *et al*., 2004). The plasmid linked gene *citP* is part of the *citQRP* operon (Magni *et al*., 1994), in which *citR* codes for a regulatory protein and *citQ* codes for a leader peptide (de Felipe *et al*., 1995; Drider *et al*., 2004). The *citQRP* operon contains promoter P1 and P2, which are located before and after an IS*982* element respectively (de Felipe *et al*., 1995). Transcription is mainly driven from promoter P1 resulting in a 2.9 kb mRNA, which is post‐transcriptionally regulated by processing in a complex secondary structure (de Felipe *et al*., 1995, 1996). Garcia‐Quintáns *et al*. (1998) showed that promoter P1 is strongly induced by low pH, which conceivably results in higher citrate uptake rates at low pH.

The effect of citrate on the expression of *citP* is still under debate (de Felipe *et al*., 1996). Harvey and Collins (1962) reported a 20 times higher citrate uptake rate of cells grown in batch cultivation on a medium supplemented with citrate compared with growth on a medium without citrate. On the other hand, Smith *et al*. (1992) did not find any significant difference in citrate uptake in a pulse experiment in chemostat cultivations on LM17 without and with supplemented citrate. Expression of *citP* and the uptake of citrate were also not affected when *L. lactis* was grown in batch cultivation on LM17 with or without supplementation of citrate (Magni *et al*., 1994).

The expression of the chromosomal *citM‐citCDEFXG* operon, containing genes encoding the citrate lyase complex and an oxaloacetate decarboxylase, as well as the citrate lyase and oxaloacetate decarboxylase activity increases at low pH (Martín *et al*., 2004; Sender *et al*., 2004). However, citrate does not affect the expression of the *citM‐citCDEFXG* operon and the citrate lyase activity in *L. lactis* (Cogan, 1981, Martín *et al*., 2004).

The aim of this study was to decipher the role of different parameters acting on overall citrate utilization capacity (i.e. uptake and metabolism) in *L. lactis* biovar diacetylactis including copy numbers of the *citP* containing plasmid, *citP* mRNA levels, *citP* mRNA processing, uptake and metabolism of citrate and quantification of metabolic end‐products such as acetoin. Bacteria were grown at constant growth rates using chemostat cultivation on chemically defined lactose‐containing media at pH 5.5 and pH 7.0, with and without supplementation of citrate under lactose and amino acid limitation.

## Results

To study the regulation of citrate uptake and metabolism, *Lactococcus lactis* biovar diacetylactis FM03‐V1 was grown in independent duplicate chemostat cultivations at a growth rate of 0.135 ± 0.004 h^−1^. In these cultivations, the bacterium was subjected to various conditions to study the effect of pH, citrate, nutrient limitation and their interactions on various cellular processes linked to citrate utilization (Table 1). In each condition, we analysed the effect on plasmid copy number of pLd1, *citP* expression, *citP* mRNA processing, citrate utilization capacity and metabolite production.

**Table 1:** Overview of the experimental design of the chemostat cultivations. All combinations of nutrient limitation, pH and citrate have been performed in duplicate in four separate cultivations. The sequences of conditions in these cultivations were as follows: cultivation 1: 1, 4, 2, 3; cultivation 2: 4, 3, 1, 2; cultivation 3 and 4: 7, 8, 5, 6. Parameters other than pH, nutrient limitation and citrate content were kept constant during the cultivations. Bacteria were grown anaerobically at a dilution rate of 0.135 h^−1^ at 30°C with a stirring speed of 300 rpm

Condition	Nutrient limitation	pH	Citrate
1	Lactose	5.5	+
2	Lactose	5.5	−
3	Lactose	7	+
4	Lactose	7	−
5	Amino acid	5.5	+
6	Amino acid	5.5	−
7	Amino acid	7	+
8	Amino acid	7	−

### Citrate utilization increases at low pH

**Figure 1:**
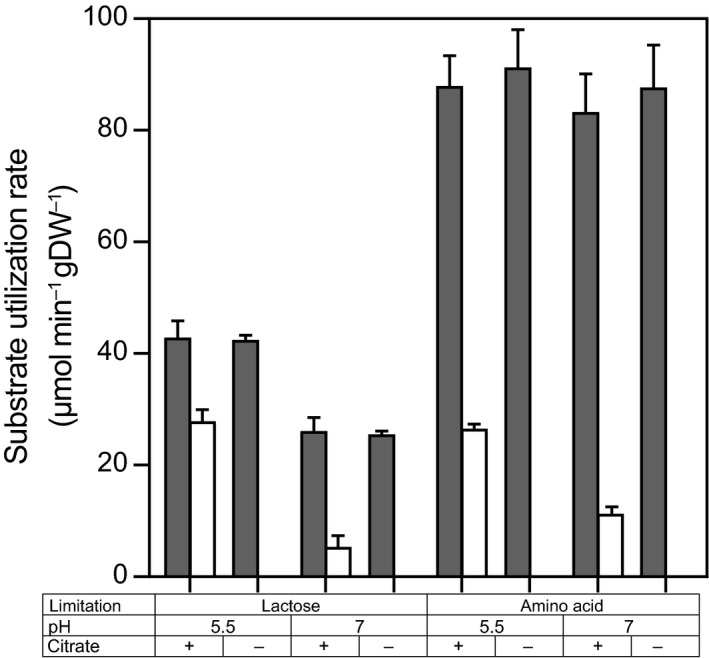
Substrate utilization rates (μmol min^−1^ gDW^−1^) of lactose and citrate during chemostat cultivation at constant growth rate (0.135 h^−1^) at different conditions: pH 5.5 or 7, in the presence (+) or absence (−) of citrate and under lactose or amino acid limitation. Filled and open bars represent lactose and citrate utilization respectively. Error bars represent the standard deviation of two biological replicates. Lactose and citrate were measured in duplicate using HPLC and enzymatic determination respectively.

The residual lactose concentration was measured to determine whether the selected media resulted in lactose and amino acid‐limited conditions indicated by complete or incomplete lactose consumption respectively. Residual lactose concentrations were below 0.05 mM and above 10 mM for lactose‐limited and amino acid‐limited media, respectively, which confirmed that growth was limited by either lactose or amino acids. In all conditions with citrate present in the medium, citrate consumption was observed. However, more than 99.7% was consumed at pH 5.5, while at pH 7, only 30% and 63% of the citrate were consumed in lactose and amino acid‐limited cultures respectively. Because the biomass concentration was similar, also the biomass‐specific citrate utilization rates were higher at pH 5.5 and under amino acid limitation (Fig. 1). These differences suggested that both an acidic environment and an amino acid limitation increased the citrate utilization capacity. To identify how these parameters regulate the citrate utilization capacity – by affecting the plasmid copy number, the *citP* mRNA level, *citP* mRNA processing or the CitP protein level – the plasmid copy number, the expression of *citP*, processing of *citP* mRNA and the citrate utilization capacity were determined.

### Higher plasmid copy number at low pH and under amino acid limitation

**Figure 2:**
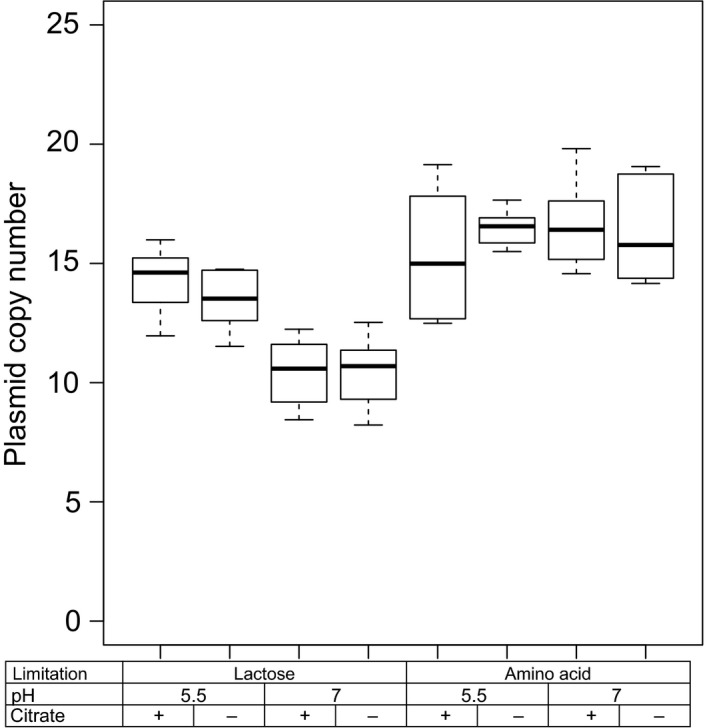
Plasmid copy number of plasmid pLd1 of chemostat grown cells cultivated at a constant growth rate (0.135 h^−1^) at different pH values, in the presence (+) or absence (−) of citrate and under lactose or amino acid limitation. The single‐copy genes *citP* and *glyA* were targeted during qPCR to quantify plasmid and chromosomal copies respectively.

To determine the plasmid copy number of plasmid pLd1, plasmid and chromosomal DNA were quantified with qPCR and the ratio between them was calculated (Fig. 2). We fitted these ratios to linear mixed effect models to find which factors significantly affected the plasmid copy number (see Experimental procedures). Significant effects were found for pH, nutrient limitation and the interaction between nutrient limitation and pH (Table 2). Compared with growth under lactose limitation at pH 7, the plasmid copy number increased both under amino acid limitation (58%) and at low pH (35%). These changes in plasmid copy numbers were caused by changes in plasmid replication rates because the growth rate was kept constant.

**Table 2:** Significance of effects of pH, nutrient limitation and the presence of citrate and their interactions on the plasmid copy number (PCN) and relative *citP* expression. Statistical analysis was performed with r by regression with a linear mixed effect (LME) model as explained in the experimental procedures. df, degrees of freedom; *t*,* t*‐value; *P*,* P*‐value

	PCN (LME)			*citP* expression (LME)		
	df	*t*	*P*	df	*t*	*P*
pH	53	−7.53	6.2 × 10^−10^	28	−2.3	0.029
Nutrient limitation	2	1.16	0.37			
Citrate				28	4.6	9.1 × 10^−5^
pH:Nutrient limitation	53	4.13	3.2 × 10^−8^			
pH:Citrate				28	−2.5	0.018

### Both citrate and low pH are required for high expression of *citP*

**Figure 3:**
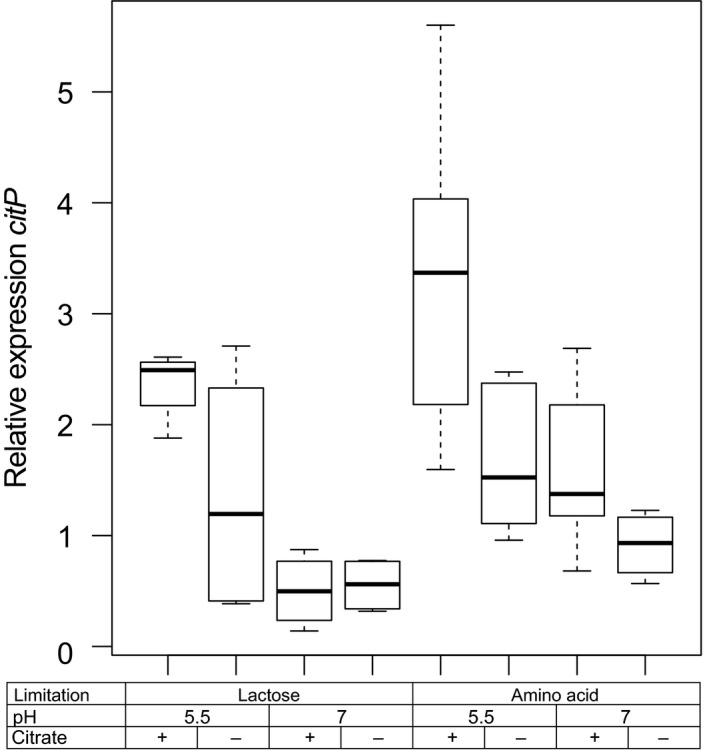
Expression of *citP* relative to the expression of the housekeeping gene *recA*. Expression has been corrected for the plasmid copy number of pLd1 (Fig. 2). Cells have been cultivated in chemostats at a constant growth rate at different pH, in the presence (+) or absence (−) of citrate and under lactose or amino acid limitation.

In addition to changes in the plasmid copy number, citrate uptake is regulated by modulating transcription of the citrate permease gene *citP*. Relative expression of *citP* was corrected for the plasmid copy numbers to quantify the effect of pH, citrate and nutrient limitation on transcription of *citP* (Fig. 3).

Fitting of the data to linear mixed effect models revealed that the pH, citrate and their interaction significantly affected the expression of *citP* (Table 2). After correction for the plasmid copy number, expression of *citP* was found to be 1.9‐fold and 2.8‐fold higher when cells were grown at low pH in the presence of citrate than when cells were grown either at low pH or in the presence of citrate respectively. No significant effect of the nutrient limitation on *citP* expression was found. This indicates that the nutrient limitation only affects the citrate utilization capacity by increasing the number of copies of plasmid pLd1.

### *CitP* processing is not affected by pH, nutrient limitation or the presence of citrate

**Figure 4:**
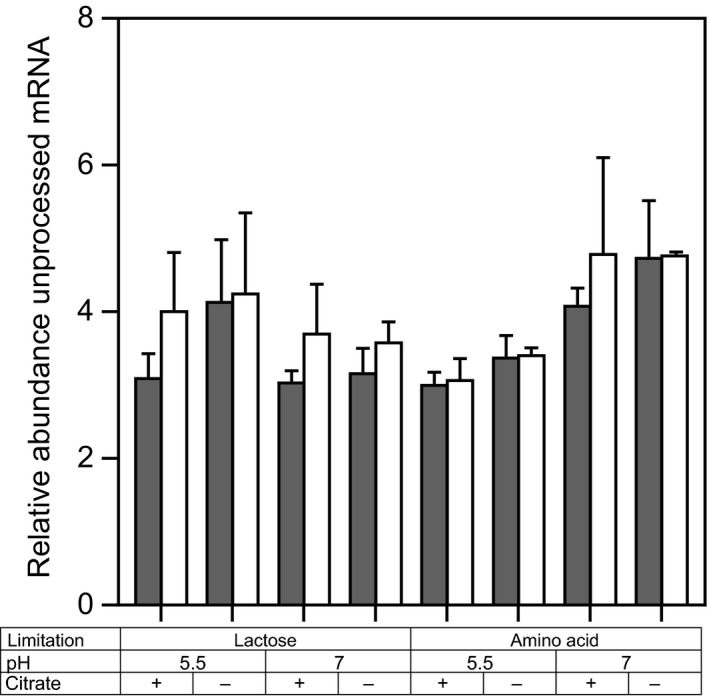
Abundance of unprocessed mRNA1 and mRNA2 (filled bars) and unprocessed mRNA1 (open bars) relative to the total *citP* mRNA (both processed and unprocessed mRNA1 and mRNA2) determined with reverse transcriptase qPCR using primers given in Table 3. mRNA1 and mRNA2 refer to mRNA produced from promoter P1 and P2 of the *citQRP* operon respectively (de Felipe *et al*., 1995). Error bars represent the standard deviation of biological duplicates.

The *citQRP* operon contains promoters P1 and P2 resulting in mRNA1 and mRNA2 of 2.9 kb and 1.9 kb respectively (de Felipe *et al*., 1995, 1996; Drider *et al*., 1998). These mRNAs are processed in a complex secondary structure (Drider *et al*., 1998). Primers sets have been designed which target specifically (i) unprocessed mRNA1, (ii) unprocessed mRNA1 and mRNA2 and (iii) both processed and unprocessed mRNA1 and mRNA2 (total mRNA; same primers as used for relative *citP* expression). To determine the effect of the pH, nutrient limitation and the presence of citrate of processing of the *citQRP* mRNA, the abundance of processed and unprocessed mRNA was analysed with reverse transcriptase qPCR (Fig. 4). Surprisingly, the PCR using primers targeting both processed and unprocessed mRNA gave a lower response than the primers targeting only unprocessed mRNA. This could be caused by processing of the mRNA at different sites than reported. Primers targeting unprocessed mRNA1 or both unprocessed mRNA1 and mRNA2 gave almost identical results showing that mRNA1 was the dominant mRNA species and transcription was mainly driven from promoter P1. No significant differences were found between the various conditions in the abundance of unprocessed mRNA1 and mRNA2 relative to the total mRNA showing that processing of the mRNAs was not affected by either pH, nutrient limitation or the presence of citrate in the medium.

### Citrate utilization capacity correlates with *citP* expression

**Figure 5:**
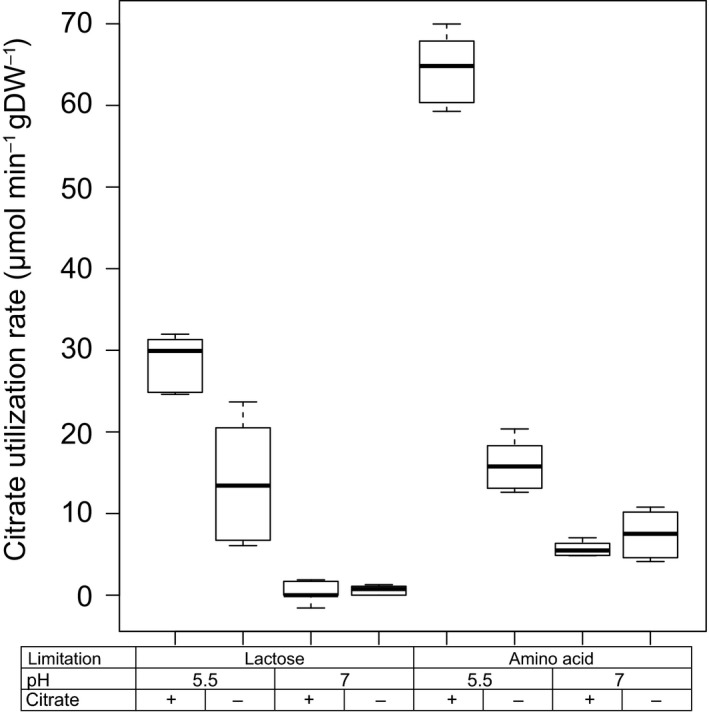
Citrate utilization capacity of chemostat grown cells cultivated at constant growth rate (0.135 h^−1^) at different pH values, in the presence (+) or absence (−) of citrate and under lactose or amino acid limitation. The rate of citrate utilization has been determined and corrected for the biomass concentration.

Expression of *citP* was compared to the citrate utilization rate to find indications of translational and post‐translational regulation mechanisms. The citrate utilization rate was determined in non‐growing cells in fresh chemically defined lactose‐free medium in which the citrate concentration was monitored in time. Similar trends were found for the citrate utilization rate as for the expression of *citP* (Fig. 5). Both low pH and the presence of citrate did significantly increase the citrate utilization rate. The measured utilization rates were similar to those found in the chemostat cultivation (compare Fig. 1 and Fig. 5). Analysis of covariance (ANCOVA) showed that none of the tested parameters significantly affected the relation between expression of *citP* and citrate utilization rate. Thus, no indication was found that citrate utilization is regulated at the translational or post‐translational level by these parameters.

### Acetoin formation increases at low pH

**Figure 6:**
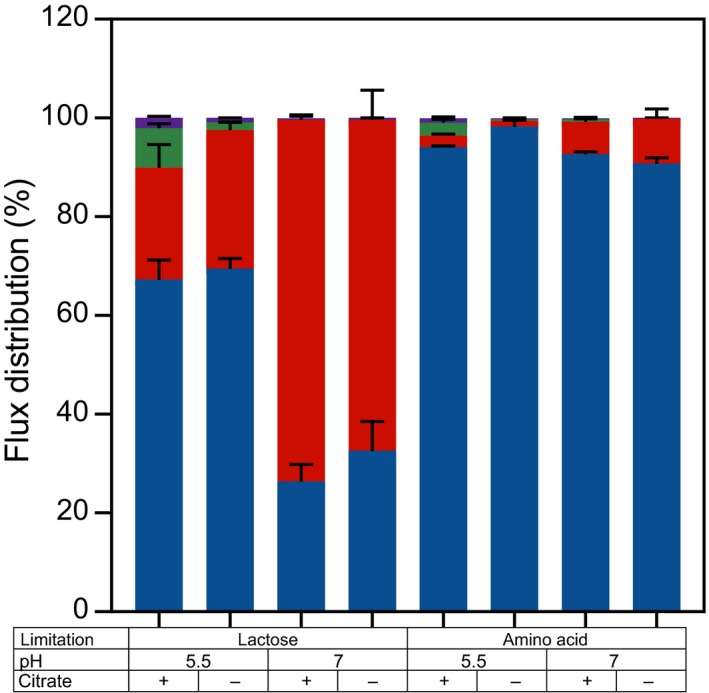
Flux distribution of the main metabolic pathways with pyruvate as substrate during chemostat cultivation at constant growth rate (0.135 h^−1^) at different conditions: pH 5.5 or 7, the presence (+) or absence (−) of citrate and under lactose or amino acid limitation. Blue: lactate dehydrogenase; red: pyruvate formate lyase; green: α‐acetolactate synthase; purple: pyruvate efflux. It has been taken into account that α‐acetolactate synthase converts two pyruvate molecules into one α‐acetolactate by multiplying the acetoin production flux by 2. Error bars represent the standard deviation of two biological replicates.

**Figure 7:**
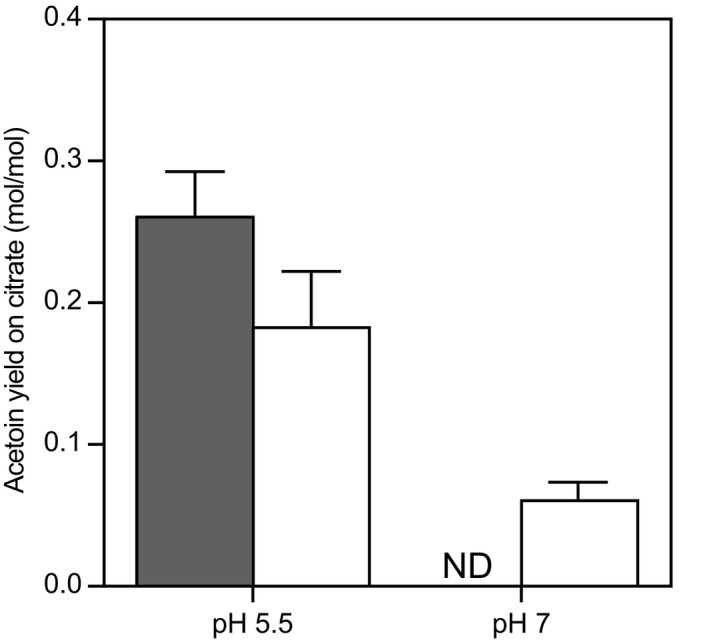
Acetoin yield on citrate (mol acetoin/mol citrate) during chemostat cultivation as function of pH and nutrient limitation. Filled bar: lactose limitation, Open bars: amino acid limitation. ND, not detected. Error bars represent the standard deviation of two biological replicates.

Although uptake of citrate was shown to be the limiting step in citrate utilization (Harvey and Collins, 1962), regulation of citrate metabolism could greatly influence the formation of flavour compounds. Metabolite production was analysed to examine the effect of pH, citrate and nutrient limitation on primary metabolism. In all cultivations, lactose and citrate were mainly converted into lactate, acetate, formate and ethanol. However, the distribution between the end‐products of fermentation differed drastically depending on the cultivation conditions (Fig. 6). Lactate was the major end‐product (> 91 mol%) under amino acid limitation independent of the pH. This metabolic profile is comparable with the profile found in batch cultures of *L. lactis* (Starrenburg and Hugenholtz, 1991). Under lactose limitation, only 68% and 29% of the end‐products were lactate at pH 5.5 and 7 respectively. Instead, a mixture of formate, acetate and ethanol was produced. Acetoin was mainly produced at low pH in the presence of citrate, but in some cases, acetoin was also produced in the absence of citrate. This indicates that citrate is the main but not the only substrate for acetoin formation. At low pH, the acetoin yield on citrate increased indicating that acetoin formation is regulated by pH (Fig. 7). Citrate is linked to acetoin formation for two reasons: (i) citrate is converted into pyruvate without production of reducing equivalents and (ii) citrate conversion leads to increased pyruvate production conceivably resulting in higher intracellular pools of pyruvate. Because α‐acetolactate synthase has a low affinity for pyruvate (K_m_ = 50 mM; Snoep *et al*., 1992), a high intracellular pyruvate concentration is required for acetoin formation. The significant export of pyruvate suggests that the intracellular pyruvate concentration was indeed high in conditions where acetoin was formed (Fig. 6).

## Discussion

**Figure 8:**
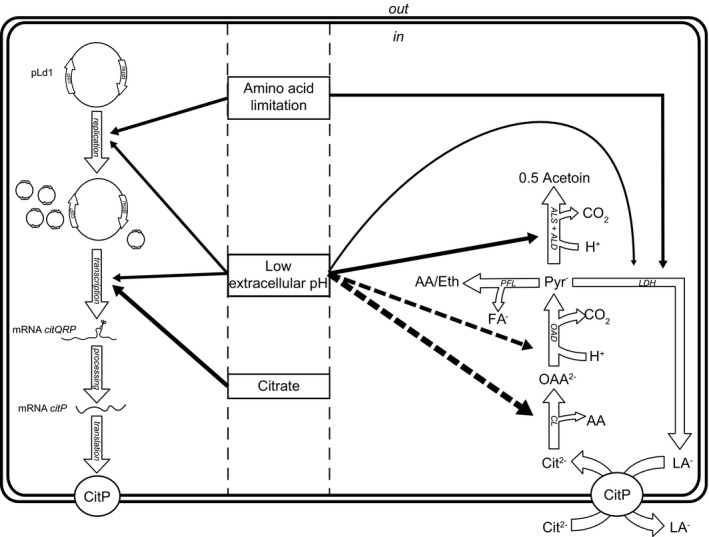
Overview of the regulation of citrate uptake and metabolism in *Lactococcus lactis* biovar diacetylactis by the three investigated parameters: pH, citrate and nutrient limitation. Filled arrows represent a positive influence on the process or reaction. Dashed arrows represent effects found in other studies (Martín *et al*., 2004; Sender *et al*., 2004). Thicker arrows represent bigger effects. On the left side, regulation of *citP* expression and translation is summarized showing regulation at the level of the plasmid copy number of pLd1 and *citP* expression. On the right side, regulation of citrate metabolism is shown. Low pH increases acetoin production via induction of citrate lyase and the acetoin forming pathway. Low pH also stimulates homolactic fermentation compared with mixed acid fermentation. Amino acid limitation mainly results in lactic acid production. Abbreviations: Cit^2‐^: divalent citrate; LA ^‐^: lactate; FA ^‐^: formate; OAA ^‐^: oxaloacetate; Pyr^‐^: pyruvate; AA: acetic acid; Eth: ethanol; CL: citrate lyase; OAD: oxaloacetate decarboxylase; ALS: α‐acetolactate synthase; ALD: α‐acetolactate decarboxylase; PFL: pyruvate formate lyase; LDH: lactate dehydrogenase.

The ability of *L. lactis* subsp. *lactis* biovar diacetylactis to metabolize citrate is an important trait for the dairy industry. In this bacterium, citrate metabolism is limited by the uptake of citrate through the citrate permease CitP (Harvey and Collins, 1962). It is known that CitP is induced at low pH (Garcia‐Quintáns *et al*., 1998), but the role of citrate is still under debate (Harvey and Collins, 1962; Smith *et al*., 1992; Magni *et al*., 1994; de Felipe *et al*., 1996). Moreover, the role of the plasmid copy number in regulating the citrate utilization capacity has never been studied. To fill these knowledge gaps, we studied *L. lactis* chemostat cultures at different conditions with the objective to elucidate the role of pH, citrate and nutrient limitation in regulation of citrate utilization. Our study revealed that amino acid limitation significantly increased the plasmid copy number of pLd1. Expression of *citP* was higher at low pH, but also the supply of citrate in the medium significantly increased transcription. An overview summarizing all factors and their interactions affecting citrate utilization is presented (Fig. 8).

The plasmid copy number of pLd1 increased approximately 1.5‐fold in cells cultivated at low pH or under amino acid limitation. The higher copy number can be ascribed to elevated plasmid replication rates because the growth rate was constant. The amino acid sequence of the replication initiation protein RepB revealed that plasmid pLd1 replicates via a theta‐type mechanism (Del Solar *et al*., 1998). At the complementary strand of the *repB* gene, a small putative counter‐transcript RNA (ctRNA) is encoded, which is located on the 5′‐end of the *repB* coding sequence (Appendix S1). This ctRNA is similar to the ctRNA of pND324 (Duan *et al*., 1998) and might also be involved in inhibition of translation of *repB* by interacting with *repB* mRNA. By this mechanism, the ctRNA could control the plasmid copy number. The role of pH and nutrient limitation in this regulation mechanism remains to be elucidated.

In addition to the effect of plasmid pLd1 copy number, we confirmed that *citP* is induced at low pH (Garcia‐Quintáns *et al*., 1998). Moreover, our study demonstrates that *citP* expression is higher in cells when citrate is present during growth and that this correlates with higher citrate utilization rates. The increased citrate utilization capacity for cells grown in the presence of citrate was also found by Harvey and Collins (1962). However, Magni *et al*. (1994) and Smith *et al*. (1992) did not find an effect on the citrate uptake rate by supplementing M17 growth medium with citrate. We demonstrated by enzymatic determination of citrate that M17 already contains 0.80 ± 0.07 mM citrate, which probably results in induction of *citP*. The presence of citrate in M17 has been overlooked by many researchers (Smith *et al*., 1992; Magni *et al*., 1994, 1999; de Felipe *et al*., 1995; Garcia‐Quintáns *et al*., 1998; Martín *et al*., 2004) and this most likely explains why those researchers did not find any effect of citrate on the regulation of citrate uptake and metabolism in *L. lactis* biovar diacetylactis.

In various heterofermentative bacteria, citrate does play a role in regulation of citrate uptake and metabolism. In *Weissella paramesenteroides*, citrate induces the *cit* operon via the transcriptional activator CitI (Martín *et al*., 2005). In addition to induction of the *citI* gene by the presence of citrate in the medium, citrate increases the DNA‐binding affinity of CitI to its two operator sites enhancing recruitment of RNA polymerase at the promoters P*citI* and P*cit* (Martín *et al*., 2005). In *L. lactis*,* citI* is induced at low pH (Martín *et al*., 2004). However, the effect of citrate on *citI* induction remains to be elucidated because all experiments in that particular study were performed with M17 grown cells. The promoter of *citI* in *L. lactis* contains an operator site for binding of CitI (Martín *et al*., 2005), and we speculate that the presence of citrate affects citrate uptake by modulating expression of *citI*. In promoter P1, which mainly drives *citP* expression, we found a putative operator site for CitI (Appendix S2). This operator site is located 121 bp upstream of the transcription start site and might be involved in regulation of *citP* by citrate via CitI.

Induction of promoter P1 results in a polycistronic mRNA containing *citQ*,* citR* and *citP* (de Felipe *et al*., 1995, 1996). *CitQ* and *CitR* are involved in post‐transcriptional regulation of CitP by processing the mRNA in a complex secondary structure (de Felipe *et al*., 1995; Drider *et al*., 1999). None of the tested parameters in this study significantly affected the processing of the mRNA or the relation between *citP* expression and the citrate utilization rate. This suggests that citrate utilization was not regulated at the post‐transcriptional level by any of these parameters. However, the amount of CitP protein has to be quantified to confirm this.

Although transport of citrate has been reported to be limiting (Harvey and Collins, 1962), enzymes responsible for citrate metabolism, that is citrate lyase and oxaloacetate decarboxylate, could become limiting under specific conditions. The genes encoding these enzymes are located on the chromosome in the *citM‐citCDEFXG* operon. Like the *citQRP* operon, this operon is induced at low pH resulting in increased activities of citrate lyase and oxaloacetate decarboxylase at low pH (Martín *et al*., 2004; Sender *et al*., 2004). Therefore, the very low citrate utilization rate at pH 7 under lactose limitation in which *citP* is expressed could be caused by low activities of citrate lyase or oxaloacetate decarboxylase.

In addition to regulating citrate uptake, pH plays a role in the metabolism of citrate. Both citrate lyase and α‐acetolactate synthase have been shown to be induced in acidic environments (Martín *et al*., 2004; Garcia‐Quintáns *et al*., 2008). These enzymes convert citrate via pyruvate into C4‐compounds acetoin and diacetyl. Therefore, induction of the genes encoding these enzymes most likely caused the increased acetoin yield at acidic conditions. Knowing the mechanisms governing citrate utilization in *Lactococcus lactis* biovar diacetylactis will help to select conditions to improve flavour formation from citrate.

## Experimental procedures

### Strain

In this study, we used *L. lactis* subsp. *lactis* biovar diacetylactis FM03‐V1. This variant has the same parent as *L. lactis* FM03, of which the genome has been sequenced (van Mastrigt *et al*., 2017). The most important difference between strain FM03‐V1, strain FM03 and their parent strain FM03P is the different plasmid contents: FM03P contains nine plasmids (pLd1 till pLd9); FM03 contains seven plasmids (pLd1 till pLd7); FM03‐V1 contains eight plasmids (pLd1, pLd2, pLd3, pLd4, pLd5, pLd6, pLd8 and pLd9). Compared with *L. lactis* FM03, FM03‐V1 shows improved lactose utilization in chemically defined medium. This is most likely caused by the presence of pLd8, which carries genes encoding PTS system for lactose uptake and the tagatose‐6‐phosphate pathway for lactose utilization. Both variants contain plasmid pLd1 that encodes the *citQRP* operon.

### Chemostat cultivation

Chemostat cultivations were carried out in duplicate in bioreactors with a working volume of 0.5 l (Multifors, Infors HT, Bottmingen, Switzerland). The temperature was maintained at 30°C, a stirring speed of 300 rpm was used and the pH was controlled at 5.5 or 7 by automatic addition of 5 M NaOH. To maintain anaerobic conditions, we flushed the headspace with nitrogen gas at a rate of 0.1 l min^−1^. Bioreactors were inoculated (inoculum size: 2% v/v) with an overnight culture in M17 (Terzaghi and Sandine, 1975) supplemented with 0.5% lactose (w/v). At the end of the exponential growth phase, continuous fresh medium supply was activated to culture the bacteria at a constant growth rate of 0.135 h^−1^ (chemostat mode). Samples were taken after reaching steady‐state conditions. A steady state was considered to be achieved after a minimum of five volume changes at which the optical density at 600 nm remained constant. The optical density was continuously monitored using an internal probe (Trucell 2, Finesse, San Jose, CA, USA). The same culture was used to analyse different conditions by changing the medium composition or the pH. Lactose and amino acid‐limited cultures were analysed in separate cultivations. Duplicate experiments of the same condition were never performed in the same cultivation.

### Media

The chemically defined media that were used differed in lactose and amino acid content to vary the type of nutrient limitation. Lactose and amino acid‐limited cultures contained per kg of medium 5.3 or 21 g lactose.H_2_O (0.5 or 2%) and 10 or 1 g Bacto‐Tryptone respectively. Citrate content was either 2.43 or 0 g kg^−1^ (NH_4_)_3_citrate. Furthermore, all media contained per kg: KH_2_PO_4,_ 2.67 g; Na.acetate.3H_2_O, 1.66 g; 100× metal stock, 10 g; 100× nucleotide stock, 10 g and 100× vitamin stock, 10 g. All media were prepared in 5L batches, adjusted the pH to 5.5 and filter‐sterilized (Millipore, Milford, MA, USA).

The 100× vitamin stock solution contained per kg: 0.2 g pyridoxine‐HCl, 0.5 g pyridoxamine‐HCl, 0.1 g nicotinic acid, 0.1 g thiamin‐HCl, 0.1 g Ca‐(D+)‐panthothenate, 1 g Na‐p‐aminobenzoate, 0.25 g D‐biotin, 0.1 g folic acid, 0.1 g vitamin B_12_, 0.5 g orotic acid, 0.5 g thymidine, 0.5 g inosine and 0.25 g DL‐6,8‐thioctic acid. pH was set at 6.8. The 100× nucleic acid stock solution contained per kg: 1 g adenine, 1 g uracil, 1 g xanthine and 1 g guanine. The compounds were dissolved in 0.1 M NaOH. The 100× metal stock solution contained per kg: 20 g MgCl_2._6H_2_O, 5 g CaCl_2_.2H_2_O, 0.5 g ZnSO_4_.7H_2_O, 0.25 g CoCl_2_.6H_2_O, 1.6 g MnCl_2_.4H_2_O, 0.25 g CuSO_4_.5H_2_O, 0.25 g (NH_4_)_6_Mo_7_O_24_.4H_2_O, 0.3 g FeCl_3_.6H_2_O and 0.5 g FeSO_4_.7H_2_O. FeSO_4_.7H_2_O was first dissolved in 10 ml 17% HCl, before it was mixed with the other compounds. All stocks were kept at −20°C until use.

### Analysis of extracellular metabolites

After taking a sample from the bioreactor, cells were directly removed by centrifugation (17 000 × *g* for 2 min at 4°C) and the supernatant was stored at −20°C until analysis. High‐performance liquid chromatography (HPLC) was performed to quantify lactose, lactate, acetate, ethanol, formate, pyruvate and acetoin on an Ultimate 3000 (Dionex, Idstein, Germany) equipped with an Aminex HPX‐87H column (300 × 7.8 mm) with precolumn (Bio‐Rad, Hercules, CA, USA). As mobile phase, 5 mM sulphuric acid was used at 0.6 ml min^−1^, and the column was kept at 40°C. Compounds were detected by a refractive index detector Shodex RI‐101 (Showa DenkoKK, Tokyo, Japan) for quantification and UV measurements at 220, 250 and 280 nm for identification.

The citrate concentration was determined with a citric acid kit (Roche, Basel, Switzerland) according to the manufacturer's procedures. The decrease in absorbance at 340 nm was measured, which correlates to the amount of NADH consumed in enzymatic reactions with citrate as substrate. This method was also used for the quantification of citrate in M17.

Extracellular metabolite concentrations were analysed in duplicate.

### Cell dry weight determination

Samples were directly taken from the bioreactor and passed through preweighted membrane filters with a pore size of 0.2 μm (Pall Corporation, Ann Arbor, MI, USA) by a vacuum filtration unit. The exact sample sizes were determined by weighing. The membrane filters were washed with at least three volumes demineralized water, dried at 80° for 48 h and weighted again on an analytical balance. Dry weights were determined in duplicate.

### Citrate utilization capacity

Citrate utilization capacity was determined in triplicate by monitoring the decrease in citrate concentration in time. Ten millilitres of cell suspension was taken from the bioreactor and directly centrifuged at 5000 × *g* for 5 min. The pellet was washed with physiological salt solution at pH 5.5, resuspended in modified chemically defined medium of pH 5.5 and incubated at 30°C. This medium was equal to the lactose‐limited medium but did not contain any lactose or acetate. Samples were taken from these cultures at regular intervals followed by direct centrifugation (17 000 × *g* for 1 min at 4°C). The supernatant was stored at −20°C until the citrate concentration was determined. In contrast to the enzymatic determination of residual citrate in chemostats, we monitored the citrate concentration using HPLC according to the protocol described before. The citrate utilization rate was calculated by dividing the change in citrate concentration in time, determined by linear regression, by the biomass concentration.

### RNA isolation

Gene expression analysis consisted of four different steps: (i) stabilization of RNA by quenching of cells, (ii) isolation of RNA, (iii) reverse transcription of RNA into cDNA and (iv) relative quantification cDNA by qPCR. A two ml cell suspension sample derived from the bioreactor was directly mixed with 4 ml RNAprotect (Qiagen, Hilden, Germany) for immediate RNA stabilization. After mixing to homogeneity, the sample was flash‐frozen in liquid nitrogen and stored at −80°C until further use. RNA was isolated using the RNeasy Mini kit (Qiagen, Hilden, Germany). Samples were thawed on ice and 1 ml sample was taken and centrifuged at 17 000 × *g* for 5 min at room temperature. The pellet was resuspended in 350 μl RLT buffer containing 10 μl ml^−1^ β‐mercaptoethanol. The suspension was transferred to a lysis matrix tube (matrix B. 0.1 mm silica spheres, Bio‐Rad, Hercules, CA, USA) and bead‐beated in three cycles of 30 s beating (4.0 m s^−1^; Bio‐Rad Fastprep‐24) and 5 min cooling on ice. Subsequently, tubes were centrifuged for 20 s (17 000 × *g*), and the supernatant was transferred to a new tube. To the supernatant, an equal volume of 70% ethanol was added, thoroughly mixed and transferred to a spin column. The different binding and washing steps are performed according to the manufacturer's procedure. To elute RNA from the column, 30 μl RNase‐free water was added and the column was centrifuged for 1 min at 17 000 × *g*. This step was repeated once. A small aliquot of the isolated RNA was used for determination of the RNA content using absorbance spectroscopy (Nanodrop; Thermo Scientific, Pittsburgh, PA, USA). The remaining sample was stored at −80°C until further use.

### Reverse transcriptase

RNA was reverse transcribed using the QuantiTect Reverse Transcription Kit (Qiagen, Hilden, Germany) according to the manufacturer's procedure. This procedure also removed remaining genomic DNA from the purified RNA samples. RNA used for the reverse transcriptase reaction was standardized at 175 ng. After the treatment, the cDNA was stored at −20°C until analysis of the expression by qPCR.

### Relative expression determined by qPCR

Relative gene expression of the citrate permease (*citP*) was quantified by qPCR (Bio‐Rad Thermal cycler CFX96‐Real‐Time system) using *recA* as reference gene. *recA* showed the most stable expression in a set of five housekeeping genes (*recA*,* tuf, gyrB*,* gyrA* and *rpoD*) during chemostat cultivation in all tested conditions (data not shown). qPCR data were analysed with bio‐rad cfx manager software. A threshold of 800 relative fluorescence unit (RFU) was used for all measurements. The qPCR reaction took place in 20 μl and contained per reaction 10 μl 2× iQ SYBR green supermix (Bio‐Rad), 0.5 μM forward and reverse primer and 1 μl cDNA. Primer sequences are given in Table 3. The qPCR program started with heating the samples for 5 min at 95°C followed by 39 cycles of 30 s at 95°C and 30 s at 58°C. Fluorescence was measured at the end of each cycle. After 39 cycles, a melting curve was made by a stepwise increase in the temperature from 65°C to 95°C in steps of 0.5°C every 5 s. To determine the efficiency of the PCR reaction, a calibration curve was made using a dilution series of isolated DNA. The efficiency did not significantly deviate from 2 for all PCR reactions (2.02 ± 0.04 and 2.05 ± 0.08). Therefore, the relative expression was calculated using an efficiency of 2 as stated in Equation (1).

(1) $$\text{Relative}\;\text{expression}=2^{C_{t,\mathrm{ref}}-C_{t,\mathrm{goi}}},$$

**Table 3:** Primer and Taqman probes used for determination of plasmid copy number, relative expression of *citP* and processing of *citP* mRNA. The gene *glyA*, located with one copy on the chromosome, was targeted in the plasmid copy number determination. *recA* was used as reference gene in the relative *citP* expression assays. Primers for *citP* were used for determination of both plasmid copy number, relative *citP* expression and *citP* mRNA processing. For the *citP* mRNA processing, *citP* primers were used to quantify both processed and unprocessed mRNA1 and mRNA2, in which mRNA1 and mRNA2 refer to mRNA produced from promoter P1 and P2 of the *citQRP* operon respectively (de Felipe *et al*., 1995)

Target		Primer sequence
*recA*	Fw primer	5′‐ACAACAGTCGCTCTTCATGC‐3′
	Rv primer	5′‐ATTTGAAGCCCTTGTTCACC‐3′
*citP* (plasmid)	Fw primer	5′‐CATTGCTATGCCAATCATGG‐3′
	Rv primer	5′‐TTCCCGAGGATAACTGTTGG‐3′
	Probe	5′‐[TXRD]CACAATACCAGCACCAACTCCACCA[BHQ2]‐3′
*glyA* (chromosome)	Fw primer	5′‐CAAAAGCAGTTATGGCAGCA‐3′
	Rv primer	5′‐ACATCAACCGCTTCTGTTCC‐3′
	Probe	5′‐[6FAM]ACGTTTCCCAGGATAACCTTCGGC[BHQ1]‐3′
Unprocessed mRNA1	Fw primer	5′‐TGAAATTAACTAGCAATTCGGGTA‐3′
	Rv primer	5′‐ACAAAGGCGCGTTTAATGTT‐3′
Unprocessed mRNA1 and mRNA2	Fw primer	5′‐CCGAGTGAAACGAGGTCAAT‐3′
	Rv primer	5′‐ACAAAGGCGCGTTTAATGTT‐3′

where *C*_t,goi_ is the threshold cycle of the gene of interest and *C*_t,ref_ is the threshold cycle of the reference gene.

Of each sample, RNA was isolated at least in duplicate and for each RNA extraction procedure the gene expression was determined by qPCR in triplicate. The average expression within one RNA extraction procedure was calculated and corrected for the measured average plasmid copy number in that specific sample. This corrected expression was used as input for the statistical analysis.

### Processing of *citP* mRNA

Processing of *citP* mRNA was analysed by reverse transcriptase qPCR as described for the determination of relative *citP* expression with the following modifications. Primers were used targeting either (i) unprocessed mRNA1, (ii) unprocessed mRNA1 and mRNA2 or (iii) both processed and unprocessed mRNA1 and mRNA2 (total RNA). The primer sequences are given in Table 3. The amount of cDNA used in the PCR reactions was decreased from approximately 175 to 87 ng. Because the efficiency of the PCR reaction was significantly lower than 2, relative abundance of the unprocessed mRNAs was calculated with equation (2).

(2) $$\text{Relative}\;\text{abundance}=\frac{E_{u}^{C_{t,u}}}{E_{t}^{C_{t,t}}},$$

where *E*_u_ and *E*_t_ are the efficiencies of the PCR reactions using primers for unprocessed mRNA species and total mRNA, respectively, and *C*_t,u_ and *C*_t,t_ are the threshold cycles of unprocessed mRNA species and total mRNA respectively.

### Plasmid copy number determination

The plasmid copy number was determined in two steps: (i) extraction of DNA and (ii) quantification of chromosomal and plasmid DNA by qPCR. DNA was extracted from bacterial cultures using the DNeasy Blood and tissue kit (Qiagen, Hilden, Germany). The amount of cells used for the DNA extraction was standardized at a very low amount (1 ml of culture with optical density of 0.02 at 600 nm) to obtain similar extraction efficiencies for chromosomal and plasmid DNA. The standardized cell suspension was centrifuged for 5 min at 17 000 × *g*. The pellet was washed with 1 ml PPS and resuspended in 0.25 ml lysis buffer (20 mM Tris‐HCl, 2 mM EDTA, 1.2% (w/v) Triton‐X‐100, 1 mg ml^−1^ lysozyme and 50 U mutanolysin, pH 8.0). The suspension was incubated at 37°C for 1 h. Subsequently, 15 μl proteinase K and 125 μl AL buffer were added. After incubation at 56°C for 1 h, 125 μl absolute ethanol was added. The released DNA was purified using a spin column provided by the kit. First, the mixture was transferred to the spin column and centrifuged for 1 min at 6000 × *g*. Then, 500 μl washing buffer AW1 was added and centrifuged for 1 min at 6000 × *g*. To remove all traces of buffer, the column was centrifuged for 4 min at 17 000 × *g*. Finally, the DNA was eluted from the column with elution buffer AE preheated at 80°C. Elution buffer was added to the column, incubated for 10 min at room temperature and centrifuged for 1 min at 6000 × *g*. This step was repeated in total three times. The extracted DNA was stored at −20°C until the plasmid copy number was determined using qPCR.

Chromosomal and plasmid DNA were quantified with qPCR (Bio‐Rad Thermal cycler CFX96‐Real‐Time system). For the chromosomal DNA control, the single‐copy gene *glyA* was used and for plasmid pLd1 the *citP* gene was targeted. Taqman probes were used to perform multiplex qPCR reactions, so PCR reactions of *glyA* and *citP* took place in a single well in 96‐wells plates. Sequences of the used primers and probes are given in Table 3. The PCR reaction took place in 20 μl and contained 0.2 mM dNTPs, 0.5 μM of all four primers, 0.25 μM of each probe, 2 μl 10× DreamTaq buffer with MgCl_2_ (Thermo Scientific, Pittsburgh, PA, USA), 0.8U DreamTaq DNA polymerase and 2 μl DNA. The PCR program started with heating the samples for 5 min at 95°C followed by 39 cycles of 10 s at 95°C and 20 s at 59°C. Fluorescence of the probes was measured. A threshold of 800 RFU was used to determine the threshold cycle (*C*_t_). An efficiency of 2 was used for both PCR reactions for calculation of the plasmid copy numbers. The following equation was used:

(3) $$\text{Plasmid}\;\text{copy}\;\text{number}=2^{C_{t,\mathrm{chromosome}}-C_{t,\mathrm{plasmid}}}.$$

From each sample, DNA was isolated at least four times, and for each DNA extraction, the PCN was determined by qPCR in sextuplicate. As input for the statistical analyses, the average plasmid copy number of a DNA extraction was taken.

### Statistical analysis

To analyse how significant the effect of the parameters (pH, citrate and nutrient limitation) was on the plasmid copy number, *citP* expression and the citrate utilization rate, statistical analyses were conducted in r (version 3.1.3) using the nlme package (version 3.1‐120 (Pinheiro *et al*., 2016)). Linear mixed effect modelling was used to correct for the nested structure of the data: different conditions were analysed in the same cultivation, while duplicates of conditions were performed in different cultivations. Therefore, cultivation was used as random categorical variable. First, the linear mixed effect (LME) model with all fixed explanatory variables (pH, citrate, nutrient limitation and their interactions) was compared with a generalized least square model (GLS) by restricted maximum likelihood estimation (REML) to see which model was better. Subsequently, the fixed explanatory variables of the selected model containing all explanatory variables were optimized by a repetitive process of fitting a full model, dropping all allowable terms in turn, comparing models by maximum likelihood estimation and dropping the least significant term. This process was repeated until all terms were significant (Zuur *et al*., 2009). The residual errors in final model were checked for homogeneity.

Analysis of covariance (ANCOVA) was used to determine whether any of the parameters (pH, citrate and nutrient limitation) had a significant influence on the relation between *citP* expression and citrate utilization rate. The average expression and citrate utilization rate per condition per cultivation was used for the analysis. A LME model with only *citP* expression as explanatory variable and cultivation as random categorical variable was compared by REML with LME models that also included pH, citrate or nutrient limitation as explanatory variable.

### Nucleotide sequence accession numbers

The sequence of plasmid pLd8 and pLd9 of *Lactococcus lactis* FM03‐V1 has been deposited at GenBank under the accession numbers MF150536 and MF150537.

## Conflict of interest

None declared.

## Supporting information


**Appendix S1**. Genetic organization of the replicon of plasmid pLd1.

**Appendix S2.** Schematic representation of *citQRP* operon in *Lactococcus lactis* subsp. *lactis* biovar diacetylactis FM03‐V1 with the putative CitI operator site.
